# Knowledge and usage patterns of clotrimazole vaginal cream and tablet amongst females: a cross-sectional study

**DOI:** 10.3389/fphar.2025.1484238

**Published:** 2025-08-08

**Authors:** Obi Peter Adigwe, Godspower Onavbavba, Chiamaka Frances Okeke

**Affiliations:** National Institute for Pharmaceutical Research and Development, Abuja, Nigeria

**Keywords:** antimicrobial resistance, rational drug use, antifungal, candidiasis, yeast infection

## Abstract

**Introduction:**

Clotrimazole is a broad-spectrum antimycotic agent that is primarily used to treat yeast and other fungal infections. Considering the limited number of medicines available for the treatment of vulvovaginal candidiasis, indiscriminate use of clotrimazole is rapidly emerging as a critical health issue. In Nigeria, where clotrimazole can be obtained without a prescription, there are public health concerns regarding the potential for antimicrobial resistance. This study therefore aimed to assess the knowledge and usage patterns of clotrimazole amongst women.

**Methods:**

A cross-sectional study involving 410 adult females in the Federal Capital Territory was conducted using a validated questionnaire. Data collected were analysed using Statistical Package for Social Sciences version 25. Descriptive statistics were used to summarise sociodemographic data, knowledge scores and usage patterns, while inferential statistics (ANOVA and t-test) examined associations between participants’ socio-demographic characteristics and knowledge levels.

**Results:**

The study included participants aged 18 years and above. Majority of the respondents learned about clotrimazole in the hospital (68.8%) and pharmacy (63.2%). Using Bloom’s cut off point, none of the participants had good knowledge of clotrimazole, and only about a quarter of the participants (26.1%) reported average knowledge regarding the use of the medicine. The overall mean knowledge score was 6.45 ± 2.43 (range 0–14). About a third of the respondents (31.7%) who had used clotrimazole in the last year reported using the medicine as a contraceptive. There was a statistically significant relationship between the level of education and participants’ knowledge score (*p* < 0.001).

**Conclusion:**

Findings from this study revealed that the participants had poor knowledge regarding clotrimazole use. Inappropriate usage patterns were also observed. These emergent findings have serious implications, particularly regarding the potential for resistance to the medicine.

## 1 Introduction

Despite limited epidemiological data, vulvovaginal candidiasis, a frequently occurring infection caused by *Candida albicans*, appears to be on the rise. This upward trend is primarily attributed to the increasing resistance of *Candida* species to widely used antifungal medications, coupled with a high recurrence rate of the infection (Hussen et al., 2024; Mohamadi et al., 2015). A 2020 study conducted in the United States reported an annual incidence of 5.2% for self-reported, healthcare provider-diagnosed vulvovaginal candidiasis (Benedict et al., 2022). The disease is not classified as sexually transmitted, and it has a tendency to affect women irrespective of their sexual status. Although *Candida albicans* is the most prevalent cause of vulvovaginal candidiasis, other *Candida* species, such as *Candida glabrata*, are becoming widely recognised as infection sources (Berman, 2012; Centers for Disease Control and Prevention, 2021).

**FIGURE 1:**
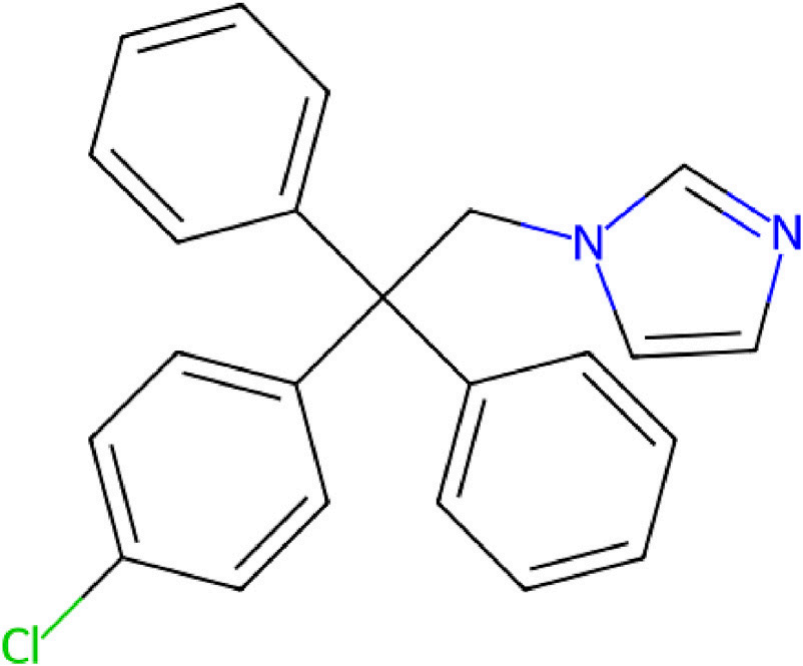
Chemical structure clotrimazole (C_22_H_17_ClN_2_).

Clotrimazole is an antimycotic agent with a broad spectrum of action belonging to the imidazole subclass of the azole antifungal family, which also includes drugs like ketoconazole and miconazole (National Library of Medicine, 2019; Kumar et al., 2021). The chemical structure of clotrimazole is shown in Figure 1. It is a lipophilic agent that acts by inhibiting lanosterol 14-α-demethylase, a cytochrome P450–dependent enzyme involved in the biosynthesis of ergosterol, an essential component of fungal cell membranes (Crowley and Gallagher, 2014). This inhibition compromises membrane integrity, leading to increased permeability and cell death (Surendran and Vidyapeetham, 2018). In addition to its antimycotic properties, clotrimazole exhibits efficacy against metronidazole-resistant trichomoniasis due to its antibacterial activity against Gram-positive organisms (Crowley and Gallagher, 2014; Midlej et al., 2019).

The antimycotic properties of clotrimazole were discovered in the late 1960s, and for decades, the drug has been marketed as a generic medicine under a variety of brand names such as Canesten and Mycoten (Kumar et al., 2021). Due to its mild side effect, metabolic profile, and fewer interactions, the drug has gained worldwide acceptance for the treatment of mycotic outbreaks such as vulvovaginal candidiasis, dermatophytosis, oropharyngeal candidiasis, and athlete’s foot (Kadavakollu et al., 2014). While generally well tolerated, topical use can cause local burning, irritation, or pruritus; allergic reactions are rare but reported (NHS, 2022). Clotrimazole is available in a variety of dosage forms which include vaginal creams, external creams, pessaries, and recently as a 10 mg oral troche (Kumar et al., 2021).

Considering the limited number of medicines available for the treatment of vulvovaginal candidiasis, the high frequency of the infection amongst women, and the multiplicity of the causative agents, the risk of resistance to clotrimazole has become a growing trend (Falahati et al., 2014). Vulvovaginal candidiasis affects an estimated 75% of women at least once in their lives, and 40% - 45% will experience two or more episodes. In one study of women over twenty-five years, about 10% had a history of vulvovaginal candidiasis, and amongst those, roughly 40% - 50% of females suffered recurrences often linked to inappropriate use of clotrimazole (Sobel et al., 2013). Vulvovaginal candidiasis is classified as uncomplicated or complicated based on clinical presentation, microbiology, host factors, and response to therapy; the complicated form occurs in approximately 10% - 20% of cases, and requires special diagnostic and treatment considerations (Centers for Disease Control and Prevention, 2021). This has therefore necessitated the use of a combination of antifungal remedies to achieve optimal synergistic effect (Shrestha et al., 2015).

To prevent resistance to clotrimazole, reducing indiscriminate use of the medicine through the multifaceted approach of antifungal stewardship is a necessary tool (Akbar et al., 2024). In Nigeria, clotrimazole is readily available over the counter. What this means is that, it can be obtained without a prescription from pharmacies as well as informal outlets such as patent medicine vendors and general shops, with little or no professional guidelines. Despite this widespread access, no study has evaluated the knowledge and usage patterns of clotrimazole in the treatment of vulvovaginal candidiasis amongst females. While clotrimazole is accessible across many LMICs, including Nigeria, there is limited published evidence assessing how women in these settings understand and use the medication, particularly outside clinical environments. This study addresses this gap by exploring real-world usage patterns and community-level knowledge, which remain underreported in similar low-resource settings. It is against this backdrop that this study aimed to assess knowledge and usage patterns towards clotrimazole.

## 2 Methods

A cross-sectional survey of adult females in Abuja was undertaken between May and July 2022. The data collection tool employed was a questionnaire (See Supplementary Material) designed in English language and developed following an extensive review of literature (National Library of Medicine, 2019; Hussen et al., 2024; Centers for Disease Control and Prevention, 2021; Berman, 2012; Midlej et al., 2019; Crowley and Gallaher, 2014; Kumar et al., 2021; Benedict et al., 2022). An iterative process involving three faculty members involved in research activities in this area was used to develop the items on the study tool. A draft version of all the items in the instrument was reviewed independently by each person, and corrections, including additions and deletions, were made. The revision process continued until a consensus was achieved. The questionnaire items were structured to gain insights into the knowledge and usage patterns associated with clotrimazole.

Face and content validation of the research instrument was undertaken by an independent expert panel, and the questionnaire was evaluated for appropriateness, complexity, attractiveness, and relevance. During the face validation, some of the statements were edited and reworded. Content validity was undertaken quantitatively; each item was tested for content validity ratio and content validity index, and only those that passed these tests were included in the final research instrument. The questionnaire was pretested to ensure that its structure could assess clotrimazole knowledge and usage patterns amongst the female population.

Using the Epi Info software version 7, a sample size of 384 was calculated for a population of 673,067 adult females in Abuja at 95% confidence level, 5% margin of error, and 50% response distribution. A stratified multistage sampling method (Parsons, 2014) was used to select the six area councils in Abuja to ensure geographical representation. Within the selected councils, adult female participants were recruited through convenience sampling at various public locations (Stratton, 2021). While this approach enabled practical data collection, it may limit the representativeness of the female population in Abuja. Nonetheless, efforts were made to include respondents across diverse age groups, occupations, and educational backgrounds to enhance the breadth and applicability of the findings.

The inclusion criteria for the study were adult females aged 18 years and above, as well as willingness to participate in the study. Participants who did not meet these requirements were not included in the study. Questionnaires were administered at various strategic locations.

Prior to the collection of data, ethical clearance was submitted to the National Institute for Pharmaceutical Research and Development Health Research Ethics Committee. Approval was received before the commencement of data collection (Approval number, NHREC/039/21A). Participation in the study was voluntary, and written informed consent was obtained from the respondents. All ethics and governance procedures were followed in accordance with the Declaration of Helsinki.

Data generated from the study were entered into the Statistical Package for Social Sciences software (SPSS) version 25. A descriptive statistical analysis was performed, and the results were expressed in frequency and percentages. For the knowledge section, correct answers were assigned 1 point, and incorrect answers assigned 0 point. The total knowledge score ranged from 0 to 14. The participants overall knowledge was categorised using Bloom’s cut-off point as good if the score was between 80% and 100% (11.2–14.0 points), moderate if the score was between 60% and 79% (8.4–11.1 points), and poor if the score was less than 60% (<8.4 points) (Seid and Hussen, 2018). To determine the relationship between variables, inferential statistical analyses were performed using ANOVA test and independent T-test. The threshold for statistical significance was *p* ≤ 0.05.

## 3 Results

### 3.1 Demography

A total of 500 questionnaires were distributed, with 410 completed and returned. About half of the study participants (48.5%) were between the ages of 18 and 29 years, with a quarter of the sample (26.6%) between the ages of 30 and 39 years. Clotrimazole was unknown to more than half of the respondents (55.1%). Other relevant details about socio-demographic characteristics are presented in Table 1.

**TABLE 1:** Socio demographic characteristics of respondents.

Variable	Frequency (%)
Age 18–29 30–39 40–49 50 and above	199 (48.5) 109 (26.6) 67 (16.3) 34 (8.3)
Educational Qualification Primary education Secondary school Diploma/NCE First Degree/HND Postgraduate level	1 (0.2) 132 (32.2) 153 (37.3) 73 (17.8) 40 (9.8)
Have you ever heard about clotrimazole? Yes No	184 (44.9) 226 (55.1)
Have you ever heard about candidiasis or yeast infection? Yes No	219 (53.4) 190 (46.3)
Have you ever had candidiasis or yeast infection? Yes No	132 (32.2) 274 (66.8)

### 3.2 Knowledge

The total mean knowledge of the respondents on clotrimazole was 6.45 ± 2.430, and with respect to Bloom’s standard, none of the participants had a score of 80% and above. A total of 73.9% of the participants had poor knowledge of clotrimazole, whilst only 26.1% had an average knowledge score.

### 3.3 Usage patterns

Findings from this study revealed that the majority of participants (89.4%) who had used clotrimazole in the last one year adhered to the prescribed dose regimen. Almost a third of the study sample (31.7%) indicated misusing clotrimazole as a contraceptive. Further details on usage patterns of clotrimazole are presented in Table 2 below.

**TABLE 2:** Usage patterns of clotrimazole.

Statement	Yes	No
	Frequency (%)	
Have you used any form of clotrimazole in the last 1 year?	104 (25.4)	300 (73.2)
If yes to the question above, did you adhere strictly to dosage instruction?	93 (89.4)	8 (7.7)
Do you think it is important to complete dosage even if all symptoms are gone?	94 (90.4)	9 (8.7)
Do you always complete your dose as prescribed by the physician?	71 (68.3)	30 (28.8)
Have you ever used clotrimazole without a prescription?	49 (47.1)	54 (51.9)
Have you ever missed a dose whilst using clotrimazole?	59 (56.7)	40 (38.5)
Do you complete the dose of clotrimazole even when you are no longer experiencing symptoms that you are treating?	92 (88.5)	11 (10.6)
Have you ever used clotrimazole as a contraceptive following unprotected sexual intercourse?	33 (31.7)	69 (66.3)

### 3.4 Source of information

**FIGURE 2:**
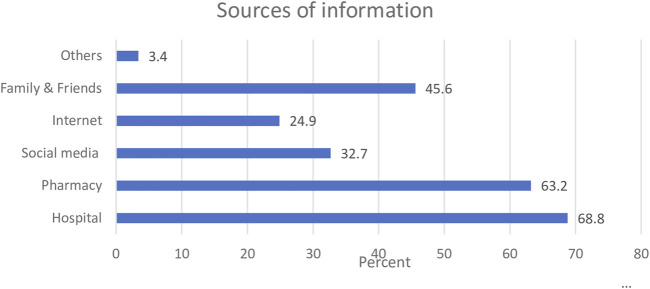
Sources of information on clotrimazole.

For the participants who had previously heard about clotrimazole, findings from the study revealed that the common source of information regarding the medicine was the hospital (68.8%), and this was closely followed by the pharmacy (63.2%). Figure 2 shows a graphical representation of the different sources of information where participants received information regarding clotrimazole.

Although hospitals and pharmacies represented the common sources of information about clotrimazole, about 45.6% of the sample indicated that they obtained information regarding the medication from family and friends.

### 3.5 Association between demographic characteristics and knowledge of clotrimazole

Inferential statistical analyses revealed that participants’ socio-demographic characteristics influenced their knowledge of clotrimazole. Participants with higher levels of education reported higher knowledge scores compared to those with lower educational levels (*p* < 0.001). Age was found to have no effect on knowledge. Further details are presented in Table 3 below.

**TABLE 3:** Association between variables and demography.

Variables	Category	Mean SD	Test of significance
Age 18–29 30–39 40–49 50 and above	a b c d	6.33 ± 2.476 6.52 ± 2.292 6.73 ± 2.416 6.47 ± 2.688	F = 0.500 (0.683)
Educational Qualification Primary education Secondary school Diploma/NCE First degree/HND Postgraduate level	a b c d e	2.00 ± 0.000 4.85 ± 1.912 6.65 ± 2.293 7.93 ± 2.043 8.20 ± 1.636	F = 30.597 (*p* < 0.001)
Have you ever heard about clotrimazole? Yes No		8.18 ± 1.720 5.05 ± 1.978	t = 16.891 (0.360)
Have you ever heard of candidiasis or yeast infection? Yes No		7.75 ± 2.035 4.94 ± 1.928	t = 14.279 (0.091)
Have you ever had candidiasis or yeast infection? Yes No		8.05 ± 1.751 5.67 ± 2.331	t = 10.390 (*p* < 0.001)

## 4 Discussion

Findings from this study revealed that the overall knowledge about clotrimazole was poor, and this could be attributed to the sources of information on the drug, as a significant proportion of the participants knew about the medicine via family, friends and other non-conventional means. Interestingly, participants who had been previously infected with candidiasis reported better knowledge on the use of the medicine compared to other respondents. This may be due to their infection-related interactions with healthcare professionals, given that two-thirds of the sample reported learn about clotrimazole from pharmacies and hospitals.

Findings from this study also revealed inappropriate use of clotrimazole, as about one-third of the participants reported using the medicine as a contraceptive following unprotected sexual intercourse. This novel finding highlights a serious impending double jeopardy in the healthcare system. Firstly, the potential development of anti-microbial resistance to clotrimazole, which is an increasing cause for concern, even globally (Akortha et al., 2009; Emeribe et al., 2015). Secondly, the risk of unwanted pregnancies associated with the use of an agent that has little or no contraceptive actions, which could have significant health and social consequences. Sources of information could have also contributed to this finding, considering the fact that close to half of the participants indicated receiving information about clotrimazole from family and friends. Available evidence has shown that obtaining information from non-professionals has significant influence on inappropriate use of medicines (Pechere et al., 2007; Calamusa et al., 2012). An increase in specific and targeted awareness campaigns will not only reduce associated antimicrobial resistance, but also ensure the correct use of indicated medicines within the system.

Almost half of the participants who had used clotrimazole for the last year indicated that they obtained the medicine without a prescription, suggesting a lack of adequate regulation in terms of access to prescription-only medicines. This can potentially increase antimicrobial resistance, which has been identified as a global challenge (Akinyandenu and Akinyandenu, 2014; McEwen and Collignon, 2018; Chukwu et al., 2021). Unaddressed, the phenomenon can further exacerbate irrational use of medicines within the Nigerian setting (Alfa and Adigwe, 2014). Interestingly, this study revealed a significant correlation between the level of education of the participants and their knowledge regarding clotrimazole use. This corroborates findings by Ross Dan Wu (1996), which reported a positive relationship between education and health. In the Nigerian setting, this finding is novel, and can underpin targeted training tools for healthcare professionals, as well as campaign materials for the general public.

More than half of the participants indicated missing at least a dose of their medication during treatment with clotrimazole, suggesting the need to intensify campaigns promoting medication adherence to the public (Hoque et al., 2020). Other initiatives that can reduce the non-adherence identified in this study include specific activation of the pharmaceutical care component of pharmacy practice, in relevant private and public sectors.

Given the high rates of non-prescription access and off-label use of clotrimazole observed, regulatory bodies should explicitly reinforce its prescription-only status by updating drug schedules and partnering with patent medicine vendor associations to monitor and curb OTC sales through routine inspections and proportionate sanctions (Oyeyemi et al., 2020). Additionally, antimicrobial resistance campaigns can incorporate public education initiatives delivered through social media platforms to address common misconceptions, such as the erroneous belief that clotrimazole can be used as a contraceptive. Finally, National AMR surveillance frameworks must be extended to include monitoring of topical antifungal sales and periodic Candida susceptibility testing, with these data feeding back into regulatory reviews.

## 5 Conclusion

This study has provided novel insights into the knowledge and usage patterns of clotrimazole amongst adult females in Abuja. The findings revealed poor knowledge about clotrimazole, with nearly three-quarters of participants scoring below the acceptable threshold. In addition, a significant proportion of participants used the medicine inappropriately, including non-prescription use and off-label application as a contraceptive. Findings from this study underscore the need for targeted education and awareness campaigns on the rational use of antimicrobial agents amongst women.

The knowledge gaps identified highlight the urgent need for pharmacists and other healthcare professionals to provide relevant information to patients, whilst also ensuring adequate follow-up that can underpin rational use of medicines and reduce non-adherence to drug therapy. Findings from this study can form a basis for further relevant research, as well as help guide policymakers and other stakeholders develop contextual policy and practice reforms that can reduce inappropriate use of antimicrobial agents.

## Data Availability

The original contributions presented in the study are included in the article/Supplementary Material, further inquiries can be directed to the corresponding authors.
