# Supplementary material for: Knowledge and usage patterns of clotrimazole vaginal cream and tablet amongst females: a cross-sectional study
Source: Front Pharmacol. 2025 Aug 8;16:1484238. doi: 10.3389/fphar.2025.1484238 (PMC12370775; doi:10.3389/fphar.2025.1484238)
Supplement: Supplementary file 1 [file Table1.docx]

**QUESTIONNAIRE**

**
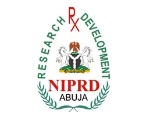
NATIONAL INSTITUTE FOR PHARMACEUTICAL RESEARCH AND DEVELOPMENT (NIPRD), ABUJA**

**Knowledge and Usage Patterns of Clotrimazole Vaginal Cream and Tablet amongst Females: A Cross Sectional Study**

**Introduction**:

Clotrimazole, also known as Canesten or Mycoten, is a broad-spectrum antimycotic agent that is primarily used to treat vaginal candidiasis and other fungal infections. This study aims at determining participants knowledge of clotrimazole as well as their usage patterns. Please fill the questionnaire by ticking (√) the most appropriate option(s). The information you provide will be treated in a completely anonymous and confidential manner.

**Section A: Demography**

1. **Age (Years)**

| 18-29 | 30-39 | 40-49 | 50 and above |
| --- | --- | --- | --- |

1. **Educational Qualification**

| Primary education | Secondary school | Diploma/NCE | First Degree/HND | Postgraduate Level |
| --- | --- | --- | --- | --- |

1. **Occupation**

| Unemployed | Student | Self-employed | Employed in Private sector | Employed in Government sector | Retired | Others, please specify ….......... |
| --- | --- | --- | --- | --- | --- | --- |

**Section B: Awareness**

1. Have you ever heard about clotrimazole?
2. Yes
3. No
4. Have you ever heard about candidiasis or yeast infection?
5. Yes
6. No
7. Have you ever had candidiasis or yeast infection?
8. Yes
9. No
10. Are you aware that improper use of Clotrimazole could be harmful?
11. Yes
12. No
13. Source of information about Clotrimazole (tick as many as possible)

| a. | Hospital |  |
| --- | --- | --- |
| b. | Pharmacy |  |
| c. | Social media |  |
| d. | Internet |  |
| e. | Family and friends |  |
| f. | Others, please specify ................................................................................................. | |

**Section C: Knowledge**

| **SN** | **Statement** | **True** | **False** | **I don’t know** |
| --- | --- | --- | --- | --- |
| 1 | Clotrimazole is an antifungal medicine |  |  |  |
| 2 | Clotrimazole can be used to treat a fungus called yeast |  |  |  |
| 3 | Clotrimazole is a prescription only medication |  |  |  |
| 4 | Clotrimazole is suitable for everyone |  |  |  |
| 5 | Clotrimazole is available as cream and vaginal tablet |  |  |  |
| 6 | Clotrimazole cream is applied 2-3 times daily |  |  |  |
| 7 | Clotrimazole vaginal tablet is used once daily |  |  |  |
| 8 | Clotrimazole should be used for a minimum of one week |  |  |  |
| 9 | Clotrimazole can be purchased from the pharmacy. |  |  |  |
| 10 | Clotrimazole can be purchased from the hospital |  |  |  |
| 11 | Clotrimazole can be purchased from the patent medicine store |  |  |  |
| 12 | Clotrimazole is also known by the brand Canesten |  |  |  |
| 13 | If Clotrimazole is not used properly, reinfection could occur |  |  |  |
| 14 | You can have sexual intercourse while on Clotrimazole |  |  |  |

**Section D: Usage Pattern of Clotrimazole**

| **SN** | **Statement** | **Yes** | **No** |
| --- | --- | --- | --- |
| 1 | Have you used any form of clotrimazole in the last one year? |  |  |
| 2 | If yes to question 25 above, do you adhere strictly to dosage instruction? |  |  |
| 3 | Do you think it’s important to complete clotrimazole dosage, even if all symptoms are gone? |  |  |
| 4 | Do you always complete your dose as prescribed by the physician? |  |  |
| 5 | Have you ever used clotrimazole without a prescription? |  |  |
| 6 | Have you ever missed a dose whilst using clotrimazole? |  |  |
| 7 | Do you complete the dose of clotrimazole even when you are no longer experiencing symptoms that you are treating? |  |  |
| 8 | Have you ever used clotrimazole as contraceptive following unprotected sexual intercourse? |  |  |

**Thank you for taking your time to complete this questionnaire**
